# Counteraction of HCV-Induced Oxidative Stress Concurs to Establish Chronic Infection in Liver Cell Cultures

**DOI:** 10.1155/2019/6452390

**Published:** 2019-02-13

**Authors:** Simona Anticoli, Donatella Amatore, Paola Matarrese, Marta De Angelis, Anna Teresa Palamara, Lucia Nencioni, Anna Ruggieri

**Affiliations:** ^1^Istituto Superiore di Sanità, Center for Gender-Specific Medicine, Rome, Italy; ^2^Department of Public Health and Infectious Diseases, Istituto Pasteur Italia-Fondazione Cenci-Bolognetti, Sapienza University of Rome, Italy; ^3^IRCSS San Raffaele Pisana, Department of Human Sciences and Promotion of the Quality of the Life, San Raffaele Open University, Rome, Italy

## Abstract

Hepatitis C virus (HCV) is a blood-borne pathogen causing acute and chronic hepatitis. A significant number of people chronically infected with HCV develop cirrhosis and/or liver cancer. The pathophysiologic mechanisms of hepatocyte damage associated with chronic HCV infection are not fully understood yet, mainly due to the lack of an *in vitro* system able to recapitulate the stages of infection *in vivo*. Several studies underline that HCV virus replication depends on redox-sensitive cellular pathways; in addition, it is known that virus itself induces alterations of the cellular redox state. However, the exact interplay between HCV replication and oxidative stress has not been elucidated. In particular, the role of reduced glutathione (GSH) in HCV replication and infection is still not clear. We set up an *in vitro* system, based on low m.o.i. of Huh7.5 cell line with a HCV infectious clone (J6/JFH1), that reproduced the acute and persistent phases of HCV infection up to 76 days of culture. We demonstrated that the acute phase of HCV infection is characterized by the elevated levels of reactive oxygen species (ROS) associated in part with an increase of NADPH-oxidase transcripts and activity and a depletion of GSH accompanied by high rates of viral replication and apoptotic cell death. Conversely, the chronic phase is characterized by a reestablishment of reduced environment due to a decreased ROS production and increased GSH content in infected cells that might concur to the establishment of viral persistence. Treatment with the prooxidant auranofin of the persistently infected cultures induced the increase of viral RNA titer, suggesting that a prooxidant state could favor the reactivation of HCV viral replication that in turn caused cell damage and death. Our results suggest that targeting the redox-sensitive host-cells pathways essential for viral replication and/or persistence may represent a promising option for contrasting HCV infection.

## 1. Introduction

Hepatitis C virus (HCV), an RNA virus belonging to the *Flaviviridae* family, represents a major worldwide concern causing about 400,000 deaths worldwide every year [1]. HCV replication cycle takes place into the cytoplasmic compartment of hepatocyte, and it causes acute or chronic hepatitis. The persistent HCV infection is clinically characterized by lifelong low-level virus production, and it is accompanied by the development of chronic liver infection (in about 80% of infected patients) that can evolve to steatosis, fibrosis, cirrhosis, and in a small percentage (about 20%) of chronically infected patients it can develop to the end-stage hepatocellular carcinoma [2].

Although the exact molecular mechanisms underlying the HCV-related liver injury are not fully understood, redox alterations of hepatocytes have been extensively described in several chronic liver diseases [3, 4]. Oxidative stress, an imbalance between the reactive oxygen species (ROS) production and their clearance by scavenging molecules, has been recognized as a leading factor in inducing hepatocyte death, inflammation, and fibrogenesis, which are responsible for induction and perpetuation of liver damage [5]. Several authors report a rise of ROS levels during HCV infection [6–13], and various viral proteins are known to induce and/or augment the ROS production, including HCV core, E1, E2, nonstructural (NS) 3, NS4B, and NS5A [11, 14–17]. Moreover, the simultaneous induction of several ROS-producing pathways and enzymes, such as the endoplasmic reticulum (ER) oxidoreductases [15, 18] and NADPH (nicotinamide adenine dinucleotide phosphate) oxidases (NOXs) [15, 16, 19], also contributes to HCV-induced oxidative stress. On the contrary, other studies report an increase in the antioxidant defenses, such as superoxide dismutase (SOD), peroxiredoxin (PRDX), glutathione S-transferase (GST) enzyme activity, and GSH levels [14, 20–23]. Glutathione is an important radical scavenger that directly and indirectly neutralizes a variety of reactive molecules, such as superoxide anions (O_2_^·−^), hydroxyl radicals, and hydrogen peroxide (H_2_O_2_) [24]. The ratio between reduced (GSH) and oxidized (GSSG) form of GSH is considered an important indicator of the antioxidant capacity of the cell. Conflicting results are shown about the effect of HCV on intracellular GSH metabolism [17, 19, 23, 25–27]. Indeed, Roe and collaborators [27] report a significant raise of GSSG in HCV-infected cells, while increased GSH concentration has been demonstrated by de Mochel et al. [19] using the same *in vitro* infection system. Interestingly, Abdalla et al. [20] describe the different effects of two viral proteins on cell antioxidant defenses. In fact, hepatocytes overexpressing HCV core protein have reduced GSH levels and increased the oxidation of thioredoxin (Trx), while the overexpression of viral NS5A protein (known for its ability to cause oxidative stress) [16] increases antioxidant enzymes (MnSOD and catalase), heme oxygenase-1 (HO-1), and GSH content. Finally, patients with chronic hepatitis C show a depletion of GSH content, which increases after antioxidant treatment [28]. However, different genotypes of HCV exhibit different abilities to induce oxidative stress [29]. In fact, in patients chronically infected with genotype 1a/b, a sharp decrease of reduced GSH level has been observed with respect to the other genotypes, suggesting the more serious disease associated with this genotype [29].

Interestingly, the de novo synthesis of GSH is controlled by the transcription factor Nrf2 (NF-E2-related factor 2), which regulates the expression of cytoprotective genes. Some studies demonstrate that acute HCV infection is associated with an early induction of proteins functioning in cellular stress responses, including the Nrf2-mediated oxidative stress response [23, 25]. On the other hand, HCV core impairs the Nrf2/ARE signaling pathway by inducing the translocation of sMaf proteins in the cytoplasm, where they bind to NS3 as part of the replication complex. As a result, Nrf2 is trapped in the cytoplasm and is unable to function as transcription factor [26, 30].

On the basis of the conflicting results reported in the literature about the relationship between redox balance and HCV infection, the goal of this study was to clarify whether the virus is able to condition the redox environment of infected cells in order to induce acute infection or to establish its persistence into the cells. For this reason, we set up an *in vitro* system that mimics as closely as possible the acute and chronic HCV infection.

Here, we demonstrate that during the acute phase of infection HCV replicates with a high rate, thus inducing intense apoptosis in infected cells; in this phase, a marked oxidative stress was registered mainly due to NOX4 activity and to a decrease of GSH and consequently of GSH/GSSG ratio. Conversely, during the chronic phase of infection, low level of both HCV replication and apoptosis was observed, ROS production returned to basal level, GSH content was reestablished, and the GSH/GSSG ratio shifted versus the reduced form of GSH. Moreover, the treatment of HCV-chronically infected cells with a prooxidant drug, auranofin, was able to reactivate viral replication.

## 2. Materials and Methods

### 2.1. Cell Lines

Huh-7.5 human hepatoma cells were generously provided by Dr. Charles M. Rice [31]. Cells were grown in high-glucose Dulbecco's modified Eagle's medium (DMEM) (Sigma) supplemented with 100 U/ml of penicillin, 100 *μ*g/ml of streptomycin, nonessential amino acids, and 10% fetal bovine serum (FBS) (Invitrogen) at 37°C in 5% CO_2_.

### 2.2. Virus

The virus stock used in this study was prepared as described below. The FL-J6/JFH1-5'C19RLuc 2Ubi plasmid, encoding the entire viral genome of a chimeric strain of HCV genotype 2a, J6/JFH1 [32], was kindly provided by C. M. Rice. The plasmid was linearized by XbaI digestion and treated with mung bean nuclease to remove 5′ end overhangs. The linearized DNA templates were first purified by phenol:chloroform extraction and ethanol precipitation and then transcribed with T7 RNA polymerase using a MEGAscript™ T7 kit (Ambion, Austin, TX) according to the manufacturer's protocol. For electroporation, Huh-7.5 cells were grown to 60–80% confluence, trypsinized, washed twice in cold PBS, and resuspended in cold PBS at a concentration of 2 × 10^7^ cells/ml. 0.4 ml aliquots were mixed with 10 *μ*g of *in vitro* transcribed RNA and dispensed into 0.4 cm Gene Pulser cuvettes (Bio-Rad). Samples were pulsed using a Gene Pulser apparatus (Bio-Rad, Hercules, CA) with a single pulse at 0.27 kV and 960 mF. Cells were resuspended in 20 ml of complete growth medium, plated and incubated at 37°C, 5% CO_2_, and 100% relative humidity. Cells were splitted every 3 days, and virus infectivity was measured by immunofluorescence assay as described below and expressed as TCDI_50_/ml. The culture supernatant from the 4^th^ passage was used to infect naïve Huh-7.5 cells at a multiplicity of infection (m.o.i.) of 0.2 in serum-free high-glucose DMEM. After 2 hours at 37°C, the medium was removed and replaced with complete medium. After 7 days and 2 passages, the supernatants were collected, centrifuged at 1200 rpm for 10 min at 4°C, cleared of debris through filtration on 0.45 *μ*m pore size filters, and stored at -80°C as virus stocks.

### 2.3. Cell Infection and Viral Titer Assays

Huh7.5 cells were plated, grown for 24 h, and then challenged with virus stock in serum-free medium at 0.01 m.o.i. Culture supernatants of uninfected cells served as a control (mock preparation). Cells were incubated in the presence of the virus for 6 h at 37°C. After the viral challenge, mock-infected and virus-infected cells were washed with PBS and then cultured with fresh medium containing 10% FBS.

The titer of infectious HCV was determined by the 50% tissue culture infective dose (TCID_50_) assay. Cell supernatants were serially diluted 10-fold in complete DMEM and used to infect 1 × 10^4^/well naïve Huh-7.5 cells in 96-well plates (8 wells per dilution). The level of HCV infection was determined three days postinfection by immunofluorescence staining for HCV core protein. The wells showing positive staining for HCV core protein were counted, and the TCID_50_ titer was interpolated using the Reed-Muench method [33].

### 2.4. Cell Treatments

Auranofin was obtained from Sigma-Aldrich (Heidelberg, Germany). The compound was dissolved in dimethylsulfoxide (DMSO) and applied to the cells at a final concentration of 200 nM that is the concentration at which no toxic effects were observed. The highest DMSO concentration present in the culture medium was 0.05%. Control cells were treated with an equivalent concentration of vehicle.

### 2.5. Cytotoxicity Assay

Cytotoxicity of auranofin was determined by cell count, and cell viability was assessed by trypan blue dye exclusion test.

### 2.6. Immunofluorescence Assay

Cells were fixed in methanol : acetone (50 : 50, *V*/*V*) for 20 min at -20°C. After blocking with PBS containing 1% bovine serum albumin (BSA) for at least 30 min, cells were incubated with mouse monoclonal antihepatitis C virus core 1b antibody (Abcam, AB 58713); bound antibodies were revealed with mouse anti-human IgG conjugated with Alexa Fluor 488 (Life Technologies, Z25102). After washing, the nuclei were stained with 1 *μ*g/ml 4,6-diamidino-2-phenylindole (DAPI; Molecular Probes) in PBS for 15 min at room temperature. Fluorescent images were acquired on an Olympus IX70 microscope equipped with Nanomover and softWoRx DeltaVision image acquisition software (Applied Precision, WA, USA) and a U-PLAN-APO 40× objective. Images were captured under constant exposure time, gain, and offset.

### 2.7. Western Blotting

Cells were detached, washed with cold PBS, and centrifuged at 700 g for 10 min. The pellet was lysed in cold RIPA lysis buffer (20 mM TRIS pH 8, 150 mM NaCl, 1% Triton-X 100, 0.1% SDS, 1% sodium deoxycholate) containing protease and phosphatase inhibitors for 30 min on ice. Lysates were centrifuged at 10 000 g for 30 min at 4°C to remove debris.

Protein concentration of cell extracts was determined by the Bradford method (Bio-Rad, 5000006). Lysates were resolved by SDS-PAGE and blotted onto nitrocellulose membranes. The membranes were blocked with 10% nonfat dry milk in Tris-buffered saline containing 0.01% Tween-100 for 1 h at room temperature (RT) and then incubated with specific primary antibodies used at final concentration of 1 *μ*g/ml. The antibodies used are as follows: rabbit polyclonal anti-NOX4 (Santa Cruz Biotechnology, sc-30141), rabbit polyclonal anti-NOX1 (Abcam, ab55831), rabbit monoclonal anti-Nrf2 (D1Z9C) (Cell Signaling Technology, Euroclone, Pero (MI) Italy, 12721S), mouse monoclonal antitubulin (Sigma-Aldrich, T5168), and mouse monoclonal anti-Lamin A/C (Sigma-Aldrich, L1293). Immunocomplexes were detected through peroxidase-coupled secondary antibodies (Jackson), followed by enhanced chemiluminescence (GE Healthcare Life Sciences). Densitometry was done using Quantity One 1-D Analysis software (Bio-Rad).

### 2.8. Glutathione Assay

The measurement of total intracellular glutathione (tGSH) (including the reduced and oxidized forms, GSH and GSSG, respectively) and GSSG was performed by means of Cayman's GSH assay kit (Cayman Chemical Co., 703002) according to the manufacturer's instructions. Briefly, the confluent control and infected Huh7.5 cells were washed twice with PBS, harvested using a rubber policeman, and were homogenized by a freeze-thaw method in 1 ml of 50 mM MES buffer pH 6 containing 1 mM EDTA. After centrifugation, a small amount of the supernatant was used for the protein assay (Bio-Rad protein assay). The residual supernatant was deproteinated by adding an equal amount of 5% *w*/*v* of metaphosphoric acid. After centrifugation (at 3000 ×g for 5 min), the supernatant was neutralized with 50 *μ*L of triethanolamine per ml of sample. The tGSH level was then determined by the endpoint method reading absorbance at 405 nm after 25 min, according to the procedures recommended by the manufacturer. In another experiment, GSH was masked by 2-vinylpyridine for 1 h before the assay to determine the GSSG levels in the samples. The tGSH and GSSG were determined by comparison with standards, normalized to protein content, and expressed as nmol/mg protein. The GSH content was obtained by subtracting the GSSG from the tGSH.

### 2.9. Cell Death Determination

The quantitative evaluation of apoptosis was performed by a double-staining flow cytometry method using fluorescein isothiocyanate- (FITC-) conjugated annexin V(AV)/propidium iodide (PI) apoptosis detection kit (Marine Biological Laboratory, MBL, Woods Hole, MA, USA), according to the manufacturer's protocol, which allows discrimination among early apoptotic, late apoptotic, and necrotic cells.

### 2.10. ROS Assay

ROS production was determined by means of the CellROX deep red reagent (Thermo Fisher, C10491), which is a fluorogenic probe, as described in the manufacturer's instructions. CellROX (5 *μ*M) was added to cell cultures and incubated at 37°C for 30 minutes. Then cells were fixed with paraformaldehyde (4%), and samples were immediately analyzed with an LRS II cytometer (Becton Dickinson, San Jose, CA, USA) equipped with a 488 argon laser and a UVB laser. The data obtained were analyzed by DIVA software (B&D). The median values of fluorescence intensity histograms were used to provide a semiquantitative assessment of ROS production. Samples were acquired with a FACScalibur cytometer (BD Biosciences) equipped with a 488 argon laser and with a 635 red diode laser. At least 20,000 events were acquired. Data were recorded and statistically analyzed by a Macintosh computer using CellQuest software (BD Biosciences). The median values of fluorescence intensity histograms were used to provide a semiquantitative assessment of ROS production.

### 2.11. Real-Time RT-PCR Analysis

Total RNA was isolated from control and HCV-infected cells, harvested at the indicated times p.i., with the RNeasy kit (Qiagen, 74104) according to the manufacturer's instructions. Isolated RNA (1 *μ*g) was reverse transcribed to cDNA using iScript™ cDNA Synthesis Kit (Bio-Rad, 1708890). A comparative real-time PCR analysis of gene expression was performed with the iQ™ SYBR Green Supermix (Bio-Rad, 170-8880) and analyzed on LightCycler iQ™ 5 (Bio-Rad). The following forward and reverse primers were used: NOX4 (5′-CAGGAGAACCAGGAGATTGTTG-3′ forward; 5′-GAAGTTGAGGGCATTCACCAGATG-3′ reverse), GR (5′-TTCAGTTGGCATGTCATC-3′ forward; 5′-CCGTGGATAATTTCTATGTGA-3′ reverse), GS (5′-GTGCTACTGATTGCTCAA-3′ forward; 5′-ACATGGATCTTCCTGTCT-3′ reverse), GCL (5′-AAGTCCCTCTTCTTTCCA-3′ forward; 5′-CCTTGAATATTGGCACATTG reverse), and GAPDH (5′-TGCGACTTCAACAGCAACTC-3′ forward; 5′-ATGTAGGCCATGAGGTCCAC-3′ reverse).

The housekeeping glyceraldehyde-3-phosphate dehydrogenase (GAPDH) was used for normalization. Relative quantitative evaluation was performed by the comparative ΔΔCt method. The results are presented as fold increase relative to control cells. Dissociation curves were generated to ensure a single amplicon had been produced.

The production of intracellular viral RNA was assessed by quantitative one-step RT-PCR carried out using an ABI 7000 Real-Time PCR System (Applied Biosystems). 5 *μ*l of total RNA was mixed with 2x TaqMan One-Step RT-PCR Master Mix (ABI) and 1 *μ*M forward (TCCCGGGAGAGCCATAGT), 1 *μ*M reverse primers (CCCAGTCTTCCCGGCAATT), and 200 nM probe (FAM- CACCGGTTCCGCAGACC -TAMRA). *In vitro* transcribed genotype 2a RNA was used as a standard to quantify the copy numbers of viral RNA.

### 2.12. Data Analysis and Statistics

A statistical significance was evaluated using GraphPad Prism™ (San Diego, CA, USA) software version 6.0. All data reported were verified in at least 3 independent experiments and are reported as means ± standard deviation (SD). Test details of each experiment are described in the figure legends. *P* values < 0.05 were considered significant.

## 3. Results

### 3.1. Generation of an *In Vitro* Model of Acute and Chronic HCV Infection

In the attempt to set up an HCV infectious cell culture system that mimics acute and chronic HCV infection, human hepatocellular carcinoma cell line (Huh7.5) was infected with HCV at low (0.01) m.o.i. to allow a multiple-cycle replication, as described in Materials and Methods, and cultured up to 76 days.

After viral adsorption, cell culture viability was monitored at different time points, and viral replication was evaluated by both real-time RT-PCR and immunofluorescence assay for HCV core protein.

**Figure 1:**
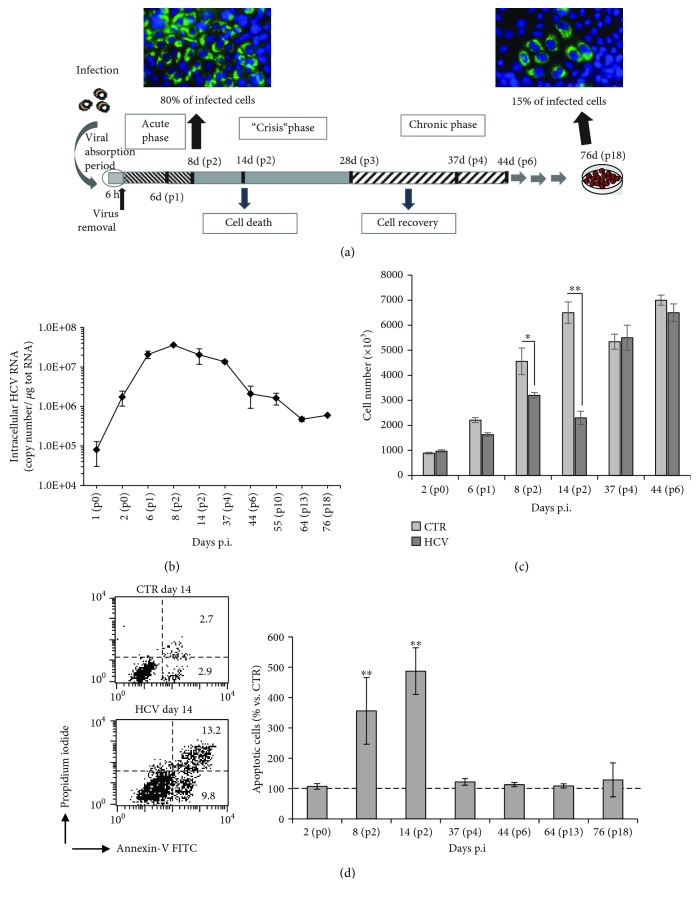
Generation of an *in vitro* model of acute and chronic HCV infection. (a) Schematic representation of the strategy to obtain long-term infected culture (LTIC). The fluorescence microscopy pictures were obtained from one representative experiment out of three performed. Cells were labelled with anti-hepatitis C virus core 1b protein followed by Alexa Fluor 488-conjugated secondary antibody (green); the nuclei were stained with DAPI (blue), 40× objective; (b) virus titration by qRT-PCR at different times of infection. (c) Trypan blue dye exclusion analysis of cell viability in uninfected (CTR) and HCV-infected Huh7.5 cells at different days postinfection. Means ± SD from three independent experiments are shown. ^∗^*P* < 0.05; ^∗∗^*P* < 0.01. (d) Biparametric flow cytometry analysis of apoptosis at different time points after HCV infection. In the left panel, a representative dot blot of apoptosis detection is shown relative to the samples obtained from 14 days p.i. Numbers represent the percentage of apoptotic cells either annexin V/PI double-positive (upper right quadrant) or annexin V single-positive (low right quadrant). In the right panel, the bar graphs show the mean ± SD of the percentage of annexin V/PI-positive cells expressed as percentage of variation vs. control uninfected cells. The mean values were obtained from four independent experiments. ^∗∗^*P* < 0.01 vs. CTR uninfected cells.

The number of viral core positive cells was estimated by immunofluorescence to evaluate the extension of HCV-infected cells. During the first two passages (8 days postinfection), about 80% of the cells were stained for HCV core protein, thus indicating a high rate of HCV replication corresponding to the “acute” phase of infection (Figure 1(a) left side). On the contrary, in the late stage of infection (76 days postinfection p.i.), here referred as the “chronic” phase, only about 15% of the cells expressed the core protein (Figure 1(a) right side). Similarly, the HCV RNA in intracellular compartment reached the highest titer (3.59 × 10^7^ ± 1.3 × 10^6^ copies/*μ*g total RNA ^∗∗∗^*P* < 0.001 vs. p0) 8 days p.i., and it decreased after the recovery phase of the cell culture and during the chronic phase (2.09 × 10^6^ ± 1.2 × 10^6^/*μ*g total RNA at 44 days p.i and 6 × 10^5^ ± 9 × 10^2^ copies/*μ*g total RNA at 76 days p.i.) (Figure 1(b)) that is consistent with a persistently infected cell culture. Furthermore, in correspondence with the maximum titer of HCV RNA, the number of HCV-infected cells was significantly reduced compared to uninfected ones (Figure 1(c)). In fact, trypan blue dye exclusion assay showed a significant decrease of alive cells during the so-called “crisis” phase that lasted about two weeks during which the infected cultures were not splitted. Particularly, on days 8 and 14 most of the cells became rounded, detached from the plate, and floated in the culture medium (data not shown). Furthermore, FACS analysis of annexin V/PI double staining of cells showed that infected cells underwent apoptotic death only during the crisis phase. In fact, as shown in Figure 1(d) (right panel), the percentage of double-stained PI+/AV+ cells increased with respect to uninfected cells (CTR) at passage 2 (p2). The 5-fold increase in percentage of apoptotic cells (obtained as mean from 4 independent experiments) was detected in infected cultures at 14 days p.i., compared to the uninfected ones (Figure 1(d), right panel). Interestingly, apoptotic death in infected cell cultures coincided with the highest intracellular viral RNA titer (Figure 1(b)), thus suggesting a causative link between the rate of HCV replication and cell death. Subsequently to the crisis phase, cells started a recovery phase at the end of which the number of live cells, in HCV-infected cultures, was comparable to that of control cells (Figure 1(c); see value at p4). Accordingly, FACS analysis demonstrated that from 37 days p.i. up to 76 days p.i., the percentage of apoptotic cells returned to control values upon recovery of the cultures (Figure 1(d) right panel; see values at p4, p6, p13, and p18). Moreover, from this time point up to 76 days p.i. (chronic phase), the percentage of HCV core-positive cells decreased to 15% (Figure 1(a) right panel) vs. 80% of the acute phase, thus mirroring viral titer trend (see Figure 1(b)). These results strongly suggested a steady state level of HCV replication.

Collectively, the results demonstrate that initial infection of Huh7.5 cells with low m.o.i. leads to obtain an acute *in vitro* infection featured by apoptotic death of infected cells, here named “acute phase”. This latter is followed by the recovery of the infected cell cultures characterized by lower virus replication and subsequently by the establishment of a persistent infection, here named “chronic phase”. All these characteristics indicated that an *in vitro* model of persistent infection with Jc1-HCV was set up.

### 3.2. HCV Infection Increases ROS Production through NOX4 and NOX1 Activity

**Figure 2:**
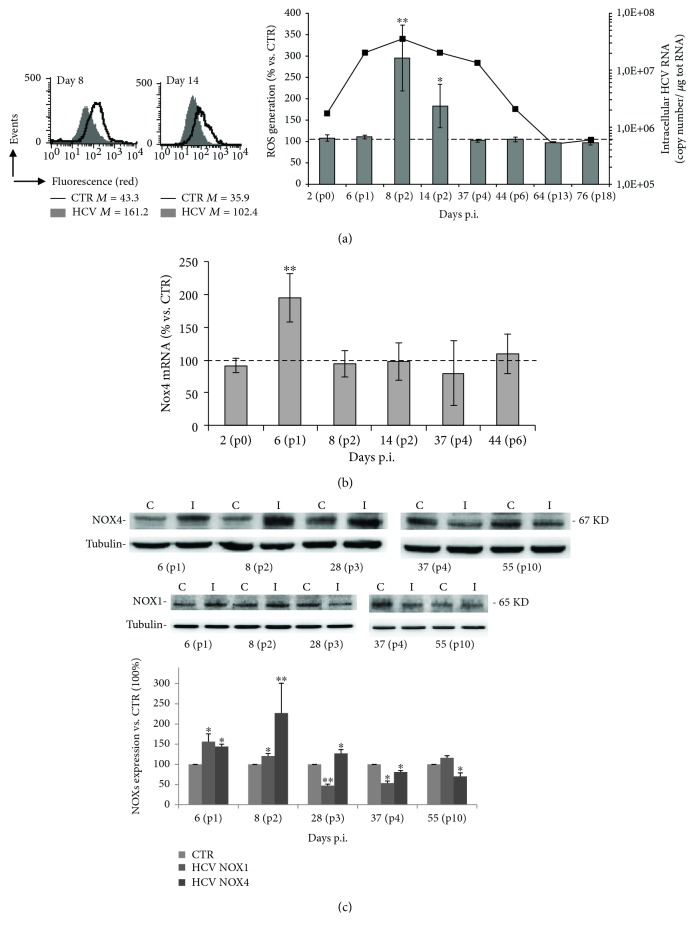
HCV infection increases ROS production through the activity of NOX4. (a) Quantitative cytofluorimetric analysis of intracellular ROS production at different time points after HCV infection in cells stained with CellROX deep red. In the left panel, data obtained from the samples relative to 8 and 14 days p.i. are shown as one representative experiment out of three performed. Numbers represent the median fluorescence intensity. In the right panel, mean ± SD of the results obtained from three different experiments are reported; the results are expressed as percentage of variation vs. CTR uninfected cells considered as 100, as indicated by the dashed line. ^∗^*P* < 0.05 and ^∗∗^*P* < 0.01 vs. CTR uninfected cells. The dark line refers to the intracellular HCV genome copy number. (b) Real-time PCR assay of NOX4 isoform levels in HCV-infected Huh7.5 cells, normalized to levels in uninfected cells (CTR), indicated with the horizontal dashed line. Data shown are the means ± SD of three performed experiments ^∗∗^*P* < 0.01. (c) Western blot of uninfected (C) and HCV-infected (I) Huh7.5 cells at different times from infection, using anti-NOX4 and anti-NOX1 antibodies. Tubulin was used as a loading control. Blot is one representative experiment out of three performed. Densitometric analysis of the blots is shown. Data represent the mean ± SD of six different technical replicates; unpaired *t* test: ^∗^*P* < 0.05 and ^∗∗^*P* < 0.01 vs. CTR (considered as 100%).

Redox changes, in particular increased ROS levels, had been associated with HCV infection [34, 35]. Nevertheless, the impact of intracellular HCV replication on cellular redox environment is so far contradictory. To this aim, confluent monolayers of Huh7.5 cells were infected with HCV, and at different time points after viral adsorption, ROS production was measured through FACS using the ROS-sensitive probe CellROX. As shown in Figure 2(a), a significant increase in intracellular ROS levels (more than 3 times higher compared to control) was detected in infected cells 8 days p.i. (p2), when HCV RNA reached the maximum value, and was maintained at high levels during all the apoptotic crisis period. After cell cultures recovery from 37 up to 76 days p.i., the ROS levels in infected cells returned to control values (Figure 2(a) right panel). These results suggest that intracellular ROS-mediated oxidative stress was induced by HCV replication, and it was simultaneously accompanied by the apoptotic phase.

Next, we investigated whether in our model the increase of ROS levels was associated with NOXs activity, particularly with two isoforms, NOX1 and NOX4, involved in the severity of HCV infection [19, 36]. To this aim, Huh7.5 cells were infected with HCV and assayed for NOX4 mRNA by RT-PCR during the different phases of HCV infection. The results demonstrated that the NOX4 mRNA levels doubled 6 days p.i. (p1) in infected cells with respect to uninfected control cells; then the NOX4 mRNA levels decreased and returned to physiological level (Figure 2(b)). Consistently, NOX4 protein expression was upregulated in infected cells during the acute phase, while it was decreased during the chronic phase (Figure 2(c)). Similar results were obtained for NOX1 enzyme, although its decrease began earlier (p3) than NOX4.

Collectively, these data suggest NOX enzymes as one possible player in HCV-induced ROS production.

### 3.3. HCV Alters the GSH Redox Homeostasis

**Figure 3:**
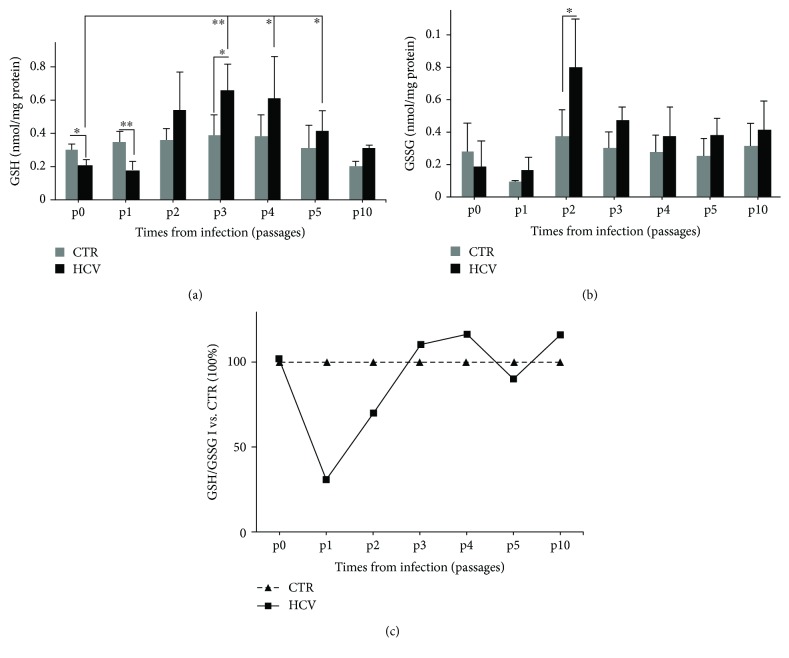
HCV alters the GSH redox homeostasis. Intracellular levels of GSH (a) and GSSG (b) in uninfected (CTR) and HCV-infected (HCV) Huh7.5 cells at different passages of infection. Data are expressed as mean ± SD of three experiments each done in duplicate (*n* = 6). 2way ANOVA followed by Sidak's multiple comparisons test: ^∗^*P* < 0.05 and ^∗∗^*P* < 0.01 I vs. CTR; ^∗^*P* < 0.05 I (p4 and p5) vs. I (p0, 2 days); ^∗∗^*P* < 0.01 I (p3) vs. I (p0, 2 days). (b) ^∗^*P* < 0.05 I vs. CTR; (c) ratio of reduced glutathione (GSH) versus oxidized glutathione (GSSG). The graph represents the ratio GSH/GSSG in infected cells (I) vs. CTR cells (considered as 100%).

To test whether Huh7.5 cells activated GSH antioxidant system in response to HCV-induced oxidative stress, the intracellular content of both reduced (GSH) and oxidized (GSSG) forms of glutathione was measured at different times after HCV infection by using a colorimetric assay. As shown in Figure 3(a), in the early steps of acute phase of infection GSH content diminished of about 40% (^∗^*P* < 0.05, ^∗∗^*P* < 0.01 at 2 (p0) and 6 (p1) days p.i, respectively) compared to uninfected cells. In contrast, the GSH levels in infected cells were significantly higher (about 70%) during cell recovery (p3) compared to the control cells and were maintained at higher levels during the chronic phase (p4, p5, and p10). More interestingly, by comparing the acute and chronic phases, a significant rise trend of GSH content in infected cells was detected in the latter phases (p3, p4, and p5 vs. p0). Conversely, as shown in Figure 3(b), the GSSG levels increased during the acute phase, in particular the content was doubled 8 days p.i. (p2). Interestingly, as shown in Figure 3(c), the GSH/GSSG ratio that under physiological conditions is maintained >1 [37] was shifted toward the oxidized form and was <1 (p1 and p2), just when viral replication reached the peak (Figure 1(b)), while during the chronic phase the ratio was >1, indicating a restore of physiological redox environment.

**Figure 4:**
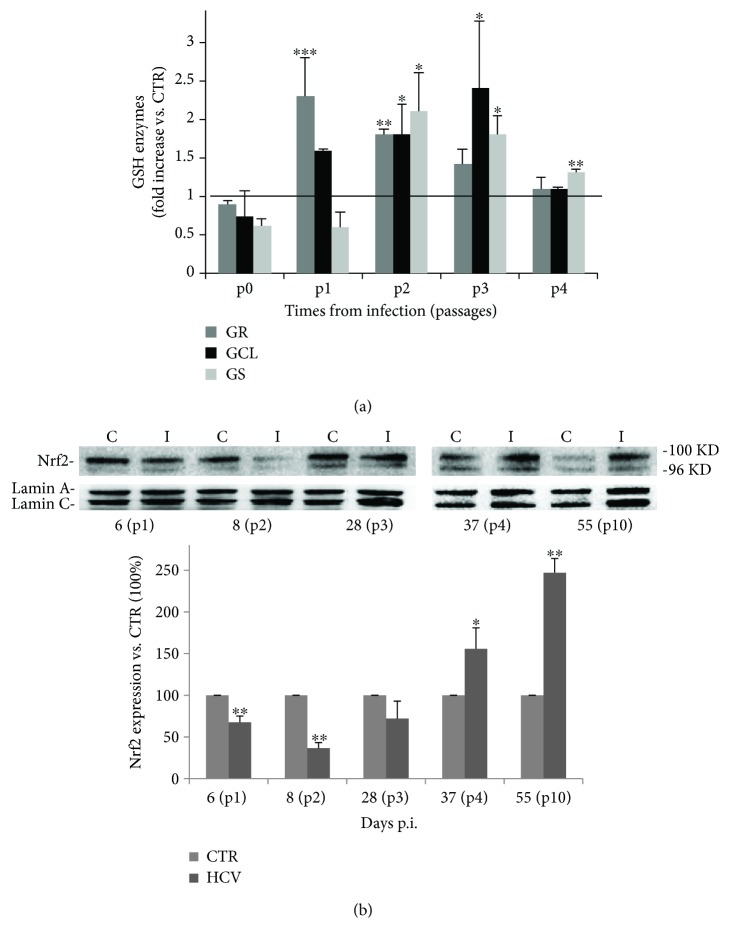
Nrf2 protein and enzymes responsible for recycling and de novo biosynthesis of GSH are differently activated during HCV infection. (a) RT-PCR quantification of enzymes responsible for recycling (GR) and biosynthesis of GSH (GCL and GS). Gene expression was measured in uninfected (CTR) and HCV-infected cells at different passages of infection. The graph represents the fold increases relative to CTR. Unpaired *t* test: ^∗^*P* < 0.05, ^∗∗^*P* < 0.01, and ^∗∗∗^*P* < 0.001 vs. CTR, represented by the horizontal line. (b) Western blot of uninfected (C) and HCV-infected (I) Huh7.5 cells at different times of infection, using anti-Nrf2 antibody. Lamin A/C was used as a loading control. Densitometric analysis of the blot is shown. Data shown are the mean ± SD of four different technical replicates; unpaired *t* test: ^∗^*P* < 0.05 and ^∗∗^*P* < 0.01 vs. CTR (considered as 100%).

Intracellular GSH is regenerated from the oxidized form by glutathione reductase (GR) or synthesized *ex novo* by the consecutive actions of glutamate cysteine ligase (GCL) and GSH synthase (GS) [38]. Therefore, we evaluated the transcriptional expression of these 3 enzymes both during the acute and chronic phase of infection. As shown in Figure 4(a), the mRNA levels of GR were strongly upregulated during the early steps of infection (p1 and p2), when the GSSG levels were high, while GCL and GS enzyme expression increased starting from the end of acute phase until the recovery period and when the peak of GSH content (p3) was detected. Interestingly, these enzymes were still activated during the chronic phase (p4), suggesting the necessity to maintain a reduced environment in long-term cultures.

Finally, we decided to evaluate whether these enzymes were under the control of Nrf2 pathway. Therefore, we analyzed Nrf2 protein expression during both the acute and chronic phases. As shown in Figure 4(b), Nrf2 seemed to be downregulated by the virus in the early phases of infection, while it was more expressed late in infection, at p4 and p10 when the chronic phase was well-established.

All these results suggest that oxidative stress during the acute phase is useful for promoting viral replication, while the reducing conditions observed in the chronic phase probably favor viral persistence into the cells.

### 3.4. Auranofin-Induced Oxidative Stress Leads to Increased Levels of HCV RNA Copies

**Figure 5:**
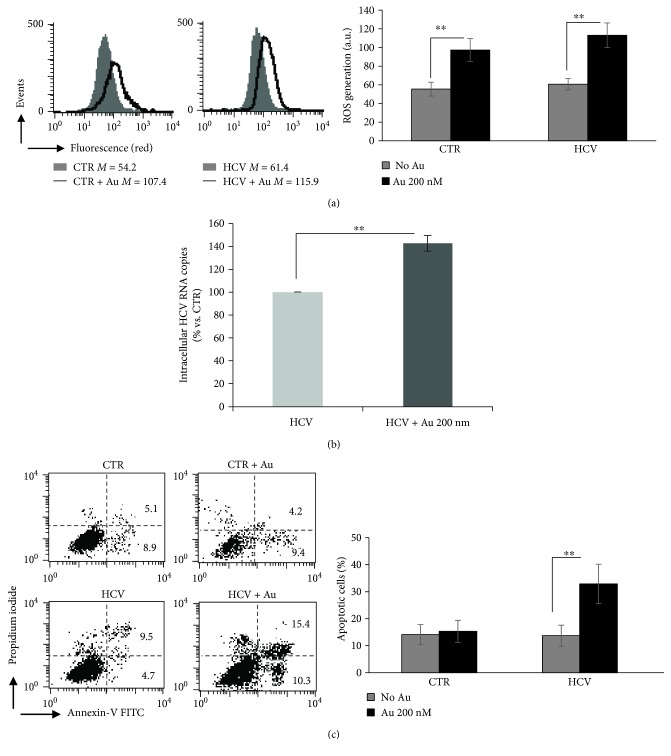
HCV induction by auranofin treatment. (a) Quantitative cytofluorimetric analysis of intracellular ROS production during the chronic phase of infection in cells treated or untreated with auranofin (Au) 200 nM and then stained with CellROX deep red. Left panel: results from one representative experiment out of three performed, are shown. Numbers represent the median fluorescence intensity. Right panel: mean ± SD of the results obtained from three independent experiments. In ordinate, median fluorescence intensity. ^∗∗^*P* < 0.01 vs. CTR uninfected cells. (b) RT-PCR analysis of HCV viral titer in the absence or presence of Au. ^∗∗^*P* < 0.01 vs. untreated infected cells. (c) Left panel: flow cytometry analysis after double staining of cells with annexin V-FITC/propidium iodide. Results from one representative experiment out of three performed, are shown. Right panel: percentage of apoptotic cells in uninfected (CTR) and HCV-infected Huh7.5 cells in the presence or absence of Au. Data are the mean ± SD of three independent experiments. ^∗∗^*P* < 0.01 vs. untreated infected cells.

Since the intracellular redox conditions seem to play a key role in the establishment of the chronic phase of HCV infection, we decided to evaluate whether the oxidative stress could reactivate HCV replication in chronically infected cells. For this reason, a chemical treatment with auranofin (Au), a well-known prooxidant drug [39] that induces ROS production in infected cells [40, 41], was used. In our experimental model, treatment with Au (200 nM) for 48 hrs was able to induce an increment of intracellular ROS levels by twofold, either in uninfected (CTR) or in HCV-infected cells (Figure 5(a)). In parallel, the number of intracellular HCV RNA copies in Au-treated cultures was about 40% greater than that of untreated infected cells (Figure 5(b)). Interestingly, in this condition, only HCV-infected cell cultures underwent apoptosis (Figure 5(c)), suggesting that oxidative stress induction could favor viral replication that in turn caused cell damage and death.

## 4. Discussion

Oxidative stress has emerged as a key contributor to the development and progression of HCV-induced pathogenesis of liver [34, 35, 42] and the associated development of hepatocellular carcinoma [43]. The lack of an *in vitro* system that recapitulates the different stages of HCV chronic infection has hampered the disclosure of the exact mechanisms and the mutual effect of HCV infection and oxidative stress pathways on the host cells. In this study, we were able to set up the HCV infectious system in Huh7.5 cells, which are permissive to the JFH1 infectious clone [31]. Particularly, infection of cell monolayers with low m.o.i. of HCV allowed to obtain first an acute infection and subsequently the establishment of a persistent infection. We demonstrated that, during the acute phase, high percentage of infected cells underwent apoptosis and viral titer peaked, whereas during the chronic phase viral replication was reduced and consequently the percentage of apoptotic-infected cells. This model called “long-term infected cultures” (LTIC) could mimic the same stages of a natural HCV infection. It is interesting that HCV has long been believed noncytopathic virus, and the hepatocyte damage during infection was considered to be mediated by the immune system. However, consistent with previously reported results [44], we could demonstrate that HCV induces cell death during *in vitro* infection of Huh7.5 cells in the absence of RIG-I-mediated innate immune response. The presence of low number of core expressing cells, during the here called “chronic phase”, paralleled by the low level viral RNA titer, indicated the ability of the JFH1 clone of HCV to persistently infect susceptible cell cultures. With regard to the mechanisms involved in establishing *in vitro* persistence, there could be a close virus-host cell interaction, and as reported by Zhong et al. [44], in Huh7.5 cells long-term infected by HCV, some cells resistant to reinfection, due to the loss of HCV receptor CD81, can be selected during the chronic phase. From the virus side, HCV is known to exist as quasispecies in patients *in vivo*, and during persistent infection *in vitro*, some virus variants can arise that are more adapted in cultures and able to persist without killing the host cells or having reduced infectivity [44]. These points will deserve deeper investigation in the future.

Furthermore, during HCV acute infection, a ROS overproduction, mainly mediated by NADPH-oxidase NOX4, was registered in infected cells. NOXs are a family of seven enzymes, generating O_2_^·−^ or H_2_O_2_ from molecular oxygen [45]. Accumulating evidence indicates that NOX-mediated ROS production has a critical role in hepatic fibrosis [46, 47], and in particular, NOX4 is involved in the severity of HCV-associated liver disease [19, 36], as well as in the regulation of viral replication of different viruses [48–50]. Here, we found that NOX4 and NOX1 rise mirrored the trend of ROS production and that of viral replication; in fact, their expression was increased during the acute phase of infection, while it decreased late in infection, thus suggesting a critical role for NOXs also in regulating HCV replication cycle. The reason why both NOX enzymes were downmodulated in infected cells during the chronic phase is still unclear, but we hypothesize that it could be a further mechanism induced by the virus to maintain reducing environment into host cell for the establishment of its persistence. Indeed, other viruses are able at the same time to positively and negatively modulate NOX isoforms to their advantage [48–50]. Further studies will be needed to clarify this aspect.

It is noteworthy that NOX4 is mainly localized in the nuclei and ER and that during HCV infection the amount of NOX4 in the nucleus increases [19], which seems to be one of the mechanisms underlying HCV pathogenesis. Therefore, it is also possible to speculate that nuclear NOX4 may increase during the acute phase of HCV infection in our experimental system, thus promoting the DNA damage and apoptosis of infected cells. Apoptotic death might favor virus spread throughout the cell culture. Further studies are in progress to deepen this aspect.

It is important to note, however, that NOXs are not the only source of ROS production in HCV-infected cells [51]. In fact, some viral proteins, i.e., core, NS3/NS4A, and NS5A, have been shown to increase ROS levels [7, 9, 52–54] through direct or indirect interaction with mitochondria. Therefore, we cannot rule out that ROS increase in HCV-infected cells might be in part produced by mitochondrial compartment, other than NOXs.

Because of high chemical reactivity of ROS, cells possess antioxidant defense mechanisms for maintaining redox homeostasis [55], whose principal component is GSH, the main abundant intracellular antioxidant. It plays a crucial role in sustaining redox balance, and the ratio of GSH to GSSG is considered as a good indicator of the redox environment. To date, there is a great debate on the induction and modulation of oxidative stress by HCV and its relationship with acute or chronic phase of infection.

Hepatocytes are the major source of GSH in the body, and the GSH antioxidant system plays an important role against oxidative stress in these cells [56]. Interestingly, reduced GSH levels were found in the serum and liver of chronically HCV-infected patients [28, 57]. Here, we found that intracellular GSH levels were significantly decreased in infected cells during the acute phase, while those of GSSG were increased, and, consequently, the ratio GSH/GSSG shifted toward GSSG, thus reflecting an impaired antioxidant potential. The oxidized environment seems to be necessary for HCV to replicate as demonstrated by viral titer peak in the acute phase. These data are in line with different *in vitro* studies on viruses responsible for acute infection, demonstrating the role of virus-induced oxidative stress in promoting viral replication [48, 50, 58–60]. On the contrary, during the chronic phase, reducing redox environment was restored by increased GSH content in infected cells that might be useful for the establishment of viral persistence. Indeed, treatment of persistently HCV-infected cells with the prooxidant drug auranofin caused the reactivation of HCV replication. The mechanisms through which HCV manipulates redox environment to its advantage are not fully clarified yet.

It is known that GSH de novo synthesis is driven by Nrf2 (nuclear factor erythroid 2-related factor), one of the major cellular defense mechanisms against oxidative stress and a master regulator of different cytoprotective genes including those involved in the antioxidant GSH pathway [61]. It has been recently demonstrated that higher expression of nuclear Nrf2 contributes to persistent HCV infection and that knockdown of Nrf2 suppresses its persistent infection [62]. The authors suggest that the nuclear translocation of p-Nrf2 might play an important role in the expression of the genes which contribute to regulate apoptosis and HCV persistence [25, 26, 30, 62]. Accordingly, we found higher mRNA levels of GCL and GS enzymes during the recovery period; at the same time, Nrf2 protein content was highly expressed during the chronic phase. On the basis of these evidences, we suggest that the high GSH levels registered in chronically infected cells compared to those measured during the acute phase of infection might be partly related to the activation of Nrf2 pathway.

There is a great debate regarding the supplementation of antioxidants in combination with antiviral drugs. In fact several clinical trials reported that antioxidant therapy decreases viral load and ameliorates hepatic damage [63]. However, other studies reported that supplementation with vitamin C, E, and selenium increased the antioxidant status but had no effects on viral load or oxidative markers [64]. All these conflicting results highlight the “dark side” of some antioxidants. Therefore, on the basis of our evidences and on the literature available, we suggest to carefully consider the use of antioxidants in chronically HCV-infected patients, since, although they could limit liver tissue injury, they would contribute to establish virus persistence that, in turn, can contribute to the development of hepatocellular carcinoma.

The lack of HCV vaccine and the emergence of viral strains resistant to antiviral therapy underline our limits in the current weapons used to fight the infection. In the recent years, new drugs acting specifically on HCV viral proteins (DAA) have been developed among which are the inhibitors of protease (telaprevir, boceprevir, and simeprevir) and the last approved RNA polymerase inhibitor, sofosbuvir [65]. Since the emergence of drug-resistant HCV variants has been recently described [66], there is an urgent need to identify novel targets for the design of new effective therapeutic strategies. In this context, targeting of redox-sensitive host-cells pathways essential for viral replication and/or persistence may represent a promising option for contrasting HCV infection.

## 5. Conclusions

In conclusion, we demonstrate that the acute phase of HCV infection is characterized by a marked oxidative stress that, similarly to other viruses, is useful for viral replication. On the contrary, the restoration of reducing redox conditions that characterize the chronic phase of infection might play a key role in decreasing viral replication and apoptotic death. Overall, our data indicate that redox sensitive pathways control the different phases of HCV infection and suggest a particular wariness in the supplementation of exogenous antioxidants in chronically HCV-infected patients for their potential role in favoring viral persistence.

## Data Availability

The data used to support the findings of this study are available from the corresponding author upon request.
