# Erratum to “Counteraction of HCV-Induced Oxidative Stress Concurs to Establish Chronic Infection in Liver Cell Cultures”

**DOI:** 10.1155/2019/3712969

**Published:** 2019-04-18

**Authors:** Simona Anticoli, Donatella Amatore, Paola Matarrese, Marta De Angelis, Anna Teresa Palamara, Lucia Nencioni, Anna Ruggieri

**Affiliations:** ^1^Istituto Superiore di Sanità, Center for Gender-Specific Medicine, Rome, Italy; ^2^Department of Public Health and Infectious Diseases, Istituto Pasteur Italia-Fondazione Cenci-Bolognetti, Sapienza University of Rome, Italy; ^3^IRCSS San Raffaele Pisana, Department of Human Sciences and Promotion of the Quality of the Life, San Raffaele Open University, Rome, Italy

In the article titled “Counteraction of HCV-Induced Oxidative Stress Concurs to Establish Chronic Infection in Liver Cell Cultures” [1], there was an error in the Authors' Contributions section. Therefore, the section should be corrected as follows:

S. Anticoli and D. Amatore equally contributed to the execution of the experiments and drafted the paper; A. Ruggieri and L. Nencioni equally contributed to conceiving the idea, designed the study, participated to the analysis and interpretation of data, and supervised the manuscript; M. De Angelis was in charge of western blotting experiments; P. Matarrese was in charge of the flow cytometry experimental analysis and interpretation of the resulting data; AT Palamara was the overall supervisor.
